# Competing risk models to estimate the excess mortality and the first recurrent-event hazards

**DOI:** 10.1186/1471-2288-11-78

**Published:** 2011-05-25

**Authors:** Aurélien Belot, Laurent Remontet, Guy Launoy, Valérie Jooste, Roch Giorgi

**Affiliations:** 1 Hospices Civils de Lyon, Service de Biostatistique, Lyon, France; 2 Université de Lyon, Lyon, France; 3 Université Lyon 1, Villeurbanne, France; 4 CNRS, UMR 5558, Laboratoire de Biométrie et Biologie Evolutive, Equipe Biostatistique-Santé, Villeurbanne, France; 5 Institut de Veille Sanitaire, Département des Maladies Chroniques et des Traumatismes, Saint-Maurice, France; 6 INSERM ERI3 « Cancers & Populations », Caen, France; 7 Registre Bourguignon des Cancers Digestifs, Inserm U866, CHU Dijon, Dijon, France; 8 Laboratoire d'Enseignement et de Recherche sur le Traitement de l'Information Médicale, EA 3283, Aix-Marseille Université, Faculté de Médecine, Marseille, France

**Keywords:** Excess hazard, Competing risks, Time-dependent hazard ratio, Regression splines, Cancer, Population-based study

## Abstract

**Background:**

In medical research, one common competing risks situation is the study of different types of events, such as disease recurrence and death. We focused on that situation but considered death under two aspects: "expected death" and "excess death", the latter could be directly or indirectly associated with the disease.

**Methods:**

The excess hazard method allows estimating an excess mortality hazard using the population (expected) mortality hazard. We propose models combining the competing risks approach and the excess hazard method. These models are based on a joint modelling of each event-specific hazard, including the event-free excess death hazard. The proposed models are parsimonious, allow time-dependent hazard ratios, and facilitate comparisons between event-specific hazards and between covariate effects on different events. In a simulation study, we assessed the performance of the estimators and showed their good properties with different drop-out censoring rates and different sample sizes.

**Results:**

We analyzed a population-based dataset on French colon cancer patients who have undergone curative surgery. Considering three competing events (local recurrence, distant metastasis, and death), we showed that the recurrence-free excess mortality hazard reached zero six months after treatment. Covariates sex, age, and cancer stage had the same effects on local recurrence and distant metastasis but a different effect on excess mortality.

**Conclusions:**

The proposed models consider the excess mortality within the framework of competing risks. Moreover, the joint estimation of the parameters allow (i) direct comparisons between covariate effects, and (ii) fitting models with common parameters to obtain more parsimonious models and more efficient parameter estimators.

## Background

Analysis of failure time data is one of the major fields of statistics, death (whatever its cause) being the event of interest. However, in some other situations, several types of events are considered and the occurrence of one type prevents the occurrence of the others, creating a context of competing risks. Initially, in that context, a subject would fail because of only one of several different event types (for example, different causes of death). However, a patient may undergo successively several event types and be considered in a situation of competing risks. For example, in a study of the efficacy of a treatment on a chronic disease, it may be interesting to analyse time to recurrence. However, a patient may present recurrence and then die or die before recurrence. This example may be analysed within the competing risks framework limiting the analysis to the first occurring event, this event being sufficient to indicate treatment failure [1,2]. When one is also interested in what happens after the first non-fatal event (e.g., recurrence), subsequent events may be seen as transitions from one state to another; this defines the framework of multi-state models [3,4]. However, in this work, we were interested in the estimation of the first-event-specific hazard functions and the covariate effects. Therefore, as in the "conventional" competing risks setting, we limited our analysis to the first occurring event whatever its type [1,4].

Our work was motivated by a dataset from a French population-based study on colon cancer patients who have undergone curative surgery. Although surgery remains the primary treatment, the incidence of recurrence after surgery increases during the first five years reaching 12.8% for local recurrence and 25.6% for distant metastasis [5]. The identification of the clinical variables associated with this treatment failure is important to determine the optimal strategy for patient follow-up, which remains controversial [6]. Moreover, patients who have undergone curative surgery and who have not experienced local recurrence or distant metastasis are still exposed to death. One might then wonder whether these patients are "cured" (i.e., their mortality has become similar to that of the general population) or still suffer excess mortality. Then, an approach based on the excess hazard model should help distinguishing expected deaths (in line with the general population mortality) from excess deaths which may be directly or indirectly related to colon-cancer [7-9].

Our objective was therefore to estimate both the excess mortality hazard and the recurrence-event hazard. Moreover, we were interested in (i) checking whether a given covariate may have the same impact on the different events, in particular on two related types of events such as "local recurrence" and "distant metastasis", and (ii) testing whether the excess mortality and recurrence-hazard are proportional or not; i.e., whether their ratio is constant over time or time-dependent in order to model it in a flexible way when appropriate. To achieve these objectives, among the many developments of the competing risk theory over the last 30 years [10-15], we used a joint modelling of the hazards associated with each type of event [4,16-19]. Indeed, the joint modelling offers, in a straightforward single analysis, the possibility to compare event-specific baseline hazards and to compare covariate effects associated with different types of events. We extended the flexible approach recently proposed to model competing risks in survival analysis [20] to the context of excess mortality and first recurrent-event hazard. Another advantage is that the model can be fitted with some of the parameters common to all event types to obtain a more parsimonious model and more efficient parameter estimators [4,18].

The paper is organized as follows. In the next section, we present our motivating example of competing risks in a prognostic study of colon cancer. Section Methods introduces the excess hazard model in case of a single event, the background of the competing risk methodology, then presents our competing risk models proposed to estimate jointly the excess mortality and the recurrent-specific hazards. In section Results, we present the analysis strategy of the motivating example and show the results. We conclude this article with a discussion of the findings and an outline of further developments.

## Motivating example

FRANCIM network is an association that joins all validated French Cancer Registries. The original dataset that stems from a "High-Resolution study" of nine French cancer registries consists of 1016 incident cases of colon cancer (caecum to rectosigmoid junction; C18 and C19 according to the International Classification of Diseases for Oncology, 3^rd ^revision) diagnosed in 1995 and treated with curative intent (surgery or endoscopic resection). Observations with unknown cancer stage at diagnosis were excluded from the analysis (n = 45). Moreover, 35 patients with synchronous distant metastasis at diagnosis (i.e., Stage IV) were also excluded because the occurrence of metastasis was one of the events under study. Thus, the analysis concerned 936 incident cases of colon cancer.

Three events were of interest: local recurrence, distant metastasis, and death. The delay from diagnosis to the first observed event was calculated in each case and patients' follow-up was restricted to the first seven years after diagnosis, time at which patients still at risk were censored. The mean age at diagnosis was 71 years (range: 21 to 100). The patients were 455 women (49%) and 481 men (51%). The cancer stages at diagnosis were 256 stage I (27%), 289 stage II (42%), and 291 stage III (31%). During the study period, there were 60 local recurrences, 143 distant metastases, and 206 deaths.

The study population presented a persistent mortality after curative intent treatment. Within such a context, an analysis of excess death with no recurrence (local or distant) should provide new insights into the course of the disease and the impact of the treatment.

## Methods

### The excess mortality hazard model

In the classical additive form, the overall mortality hazard function, *λ*_*O*_, is split into an excess hazard function, *λ*_*+*_, and a population (or expected) hazard function *λ*_*P*_(1)

where *t *is time since diagnosis, *a *the age at diagnosis, **x **a vector of covariates, and **z **a vector of population characteristics [8,9].

The population hazard function *λ*_*P*_(*a + t*, **z**) in (1) is assumed to be known and is usually quantified on the basis of a vector **z **of population characteristics (generally age, sex, and possibly place of residence, etc.) and may be obtained from national statistics institutes. In previous works, the excess hazard function was modelled by proportional hazard (PH) models *λ*_+_(*t*, **x**) = *λ*_0 _(*t*)exp(**βx**) with *λ*_0_(*t*) constant within pre-specified intervals of follow-up [8,9]. Modelling the baseline hazard function and relaxing the PH assumption were also proposed using either regression splines or fractional polynomials [21-23].

### Background of the competing risks

Competing risk data in a sample of N patients give rise to a right censored sample (*t_i_,δ_i_,j_i_*,**x**_*i*_), *i *= 1,...,*N *where *t*_*i *_is the time to the first event, *δ_i _*the status (or failure) indicator equal to 1 in subjects *i *that present any event and 0 otherwise, *j_i _*the type of event (for example, in the case of three competing events, *j_i _*= 1,2,3 and *j_i _*= 0 if the subject is censored at *t*_*i*_), and **x**_*i *_a vector of covariates.

Assuming a random censoring mechanism, the full likelihood function [10] is:(2)

where *λ_j_*(*t*,**x**) is the event *j*-specific hazard,  the overall (all-events) survival in the case of *J *distinct types of events, which quantifies the probability of surviving until time *t *without experiencing any of the *J *distinct events, and *I*(*j_i _*= *j*) is the indicator function equal to 1 for a event of type *j *observed at time *t_i_*.

When death is among the *J *event types, the event-specific hazard for death represents the mortality hazard due to all causes; i.e., the observed deaths combine "expected deaths" and "excess deaths". The excess hazard model offers an attractive approach to evaluate the event-specific hazard associated with "excess death" and to assess the effect of the covariates on the excess mortality hazard.

### New competing risk models in excess mortality analysis

#### The proposed models

The new model combining the excess mortality hazard model and the competing risk model may be written:(3)

where, in case of *K *covariates, *b*_1 _= 0 and *β*_1*k *_= 0, *k *= 1,...,*K *for uniqueness.

To simplify the interpretation of model (3), we considered *J *= 3 different events with event "death" denoted by *j *= 3 as an example. That model assumes a common pattern for the baseline hazards through *λ*_1_(*t*) which represents the baseline hazard for individuals experiencing the 'reference' event 1 and with vector of covariates **x **equal to 0. In model (3), the log of the baseline hazard of event 1, log(*λ*_1_(*t*)), is modelled by a cubic regression spline with one knot located at 1 year. The interior knot location at 1 year is suggested because, in many cancers, a large proportion of events are observed during the first year after diagnosis. However, the user may either choose another knot location, based on substantive knowledge about the disease or, in the absence of such knowledge, locate the interior knot at the median of the sample distribution of uncensored event times, which ensures equal data support for both functional segments. A cubic regression spline is a smooth piecewise polynomial function of order 4 in which the constraint is that the function and its first two derivatives should be continuous at the knots where the adjacent pieces of the polynomial join [24,25]. Since the baseline hazard for event 1 is *λ*_1_(*t*), the baseline hazard for the event *j *≠ 1 is simply *λ*_*j*_(*t*,**x **= 0) = *λ*_1_(*t*)exp(*b_j_*). For event death, the baseline hazard is *λ*_1_(*t*)exp(*b*_3_) which represents the baseline of the "event-free excess death hazard". The model (3) assumes PH effects of covariates **x **on the event-specific hazards. For one unit increase of a given covariate *x*_*k*_, the effect is split into a *common *(or shared) effect through the regression parameter *α_k_*, and a *differential *event-specific effect through the regression parameter *β_jk_*. In the same way, the HRs will be estimated by exp(*α_k_*) and exp(*α_k _*+ *β_jk_*) for events 1 and *j*, respectively. So, the comparison between covariate effects on event 1 and event *j *can be directly tested by H_0_: *β_jk _*= 0 using the classical Wald test or the likelihood ratio (LR) test. Moreover, when the effect of a covariate on one event type is not significantly different from the common effect, a simpler and more parsimonious variant of model (3) can be fitted, including only the common effect *α_k _*of this covariate.

However, the assumption of a common pattern for event-specific hazards through *λ_1_*(*t*) may seems dubious [17,20]. To overcome this limit of our new model (3), we propose to introduce a time-dependent log HR, *b_j_*(*t*), between event-specific hazards.

The new flexible model may be written:(4)

where, in case of *K *covariates, *b*_1_(*t*) = 0 and *β*_1*k *_= 0, *k *= 1,...,*K *for uniqueness.

In the flexible model (4), the log of the baseline hazard of event 1, log(*λ*_1_(*t*)), and the time-dependent log HRs between event-specific hazard, *b_j_*(*t*), are modelled by cubic regression splines, each spline having one knot located at 1 year. (Note that while all spline functions in (3) and (4) use the same cubic B-spline basis, the resulting functional estimates may differ substantially in their values and shapes, depending on the estimated spline coefficients). Then, each time-dependent effect is modelled by a 5-degree-of-freedom (df) function [26] and a LR test with 4 df can be used to compare a model with a constant *b_j _*versus a model with *b_j_*(*t*) (i.e., to compare the new model (3) to the new flexible model (4)). Therefore, in the case of non-significant time-dependent effects *b_j_*(*t*),*j *= 2...*J*, the simpler model (3) may be used. Moreover, model (4) is an important and flexible alternative in modelling because some HRs between baseline hazards may be time-dependent (e.g., death and local recurrence) whereas others may be constant over time (e.g., for events close in nature such as local recurrence and distant metastasis).

#### Estimation procedure

In both models (3) and (4), the maximum likelihood estimates are obtained using the trick of data duplication. A detailed description of data duplication and coding can be found in references [4,16,17,27]. This trick allows fitting both models (3) and (4) using any tool for one survival outcome existing in statistical software, such as the Cox model [17]. In the present work, the maximum likelihood estimates are obtained using an Iterative Reweighted Least Square procedure developed for a previous spline-based model [23]. This procedure is based on split data, which approximates the contribution of each individual to the full log-likelihood by a sum of Poisson terms on time intervals that are sufficiently small for the assumption of a constant rate to be acceptable [23,28]. Doing so, the parameters can be estimated within the framework of the generalized linear models assuming a Poisson distribution for the observed number of deaths. However, as pointed by Dickman *et al.*, the user has to specify a particular link function for the generalized linear model to take into account the general population mortality in the estimation procedure [29].

#### Performance of the estimators

Simulation studies were conducted to assess the performance of the estimators obtained from model (4) in the case of three competing events (of whom death) and different sample sizes and censoring rates. Data generation, simulation design, and results are detailed in Additional file 1.

Briefly, the times to the events were supposed to depend on three independent prognostic factors. Different rates of drop-out censoring (0%, 15%, and 30%) and different sample sizes (N = 400 and N = 1000) were considered. The relative biases (RBs) were close to zero (range: -0.047 to 0.05) whatever the sample size and the drop-out censoring rate (Additional file 1, Table S1). Obviously, the RBs increased with the drop-out censoring rate for most parameter estimates and the impact of the drop-out censoring rate was more important with N = 400 than with N = 1000. Whatever the sample size and the drop-out censoring rate, the empirical coverage rates (ECRs) were close to the nominal level of 95% (range: 91.8 to 96.6), even when the ECR was slightly smaller than 95% for the parameter estimates of the excess mortality hazard function (Additional file 1, Table S1). Graphically, we have shown that the means of the estimates of the baseline hazard function of the three competing events were close to their true baseline hazard functions (Additional file 1, Figures S1 and S2). The performances of the estimated time-dependent HRs relative to event 2 and event 3 (excess death) were similar.

## Results

### Analysis strategy

In this analysis, our objectives were: (i) estimate the baseline hazards, and their ratios, for local recurrence, distant metastasis, and recurrence-free excess death; (ii) to estimate the effects of sex, age at diagnosis, and stage at diagnosis associated with each event-specific hazard; and (iii) to test whether the effects of covariates are common to the two related events "local recurrence" and "distant metastasis" or not.

The strategy consisted of three steps. The first step was to determine the pattern of the time-dependent HRs between event-specific hazards. Thus, a Cox PH model with constant HRs between event-specific hazard functions was used on duplicated data; i.e., with a dummy variable denoting each type of event introduced as covariate [4,17]. In this model, the baseline hazard function was the baseline hazard function for local recurrence. Using Schoenfeld residuals, the pattern of the time-dependent HR was obtained graphically [30]. The second step was to estimate the baseline hazard for local recurrence. Considering only local recurrences as events and censoring the other events, we fitted six models using six candidate functions of time: constant, linear, quadratic polynomial, cubic polynomial, regression cubic spline with one knot at 1 year, and regression cubic spline with two knots at 1 and 5 year. The final baseline hazard for local recurrence was selected with the Akaike Information Criterion (AIC) [31]. The third step was to jointly estimate and test the covariate effects with the flexible model (4), using the time-dependent HR obtained from the first step and the pattern of the baseline hazard for local recurrence obtained from the second step. In the tests described below, we imposed different covariate effects on the death event than on the two others, and the test for a given covariate was performed adjusted on the other covariates. More precisely, to test whether a covariate x had a different effect on "local recurrence" than on "distant metastasis", we compared a version of model (4) with a common effect on "local recurrence" and "distant metastasis" against another version of model (4) with different effects on "local recurrence" and "distant metastasis" using a LR test (with 1 df for covariates sex and age and 2 df for covariate stage). For this analysis, the population mortality hazard function was obtained from the French vital statistics published by the Institut National de la Statistique et des Études Économiques (INSEE), detailed by sex and age.

### Results of the analysis

At the first step of our strategy, it was obvious to use a constant hazard ratio of the baseline hazards of distant metastasis event to that of local recurrence event, while a cubic regression spline with one knot at one year was used to model the time-dependent HR of the death event to that of local recurrence event.

**Figure 1:**
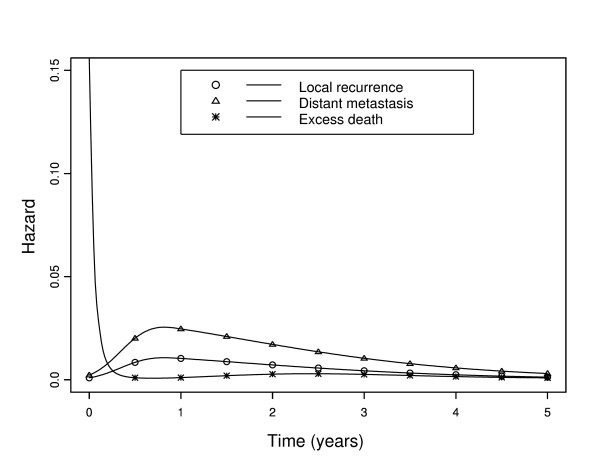
**Event-specific baseline hazard functions for local recurrence, distant metastasis, and excess death in 936 French colon cancer patients**.

**Figure 2:**
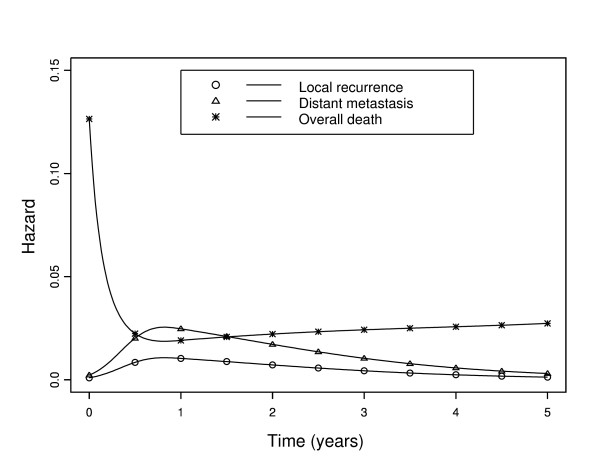
**Event-specific baseline hazard functions for local recurrence, distant metastasis, and overall death in 936 French colon cancer patients**.

At the second step, the AIC approach selected a regression cubic spline with one knot at 1 year to model the baseline hazard relative to the local recurrence event. As shown in Figure 1, the baseline hazards relative to the local recurrence event and the distant metastasis event increased to a maximum during the first year after diagnosis and then decreased slowly. However, the baseline hazard relative to the distant metastasis event was higher than that of the local recurrence event. The baseline (recurrence-free) excess death hazard was very high just after diagnosis, probably due to post-surgical complications; it sharply decreased thereafter until six months then reached a constant value close to zero. In absence of post-surgical complications, operated patients were exposed to the same risk of death as the general population as long as they did not relapse. Not taking into account the expected mortality may lead to an erroneous conclusion about the benefit of surgery. To verify this, we performed an analysis with a simplified version of the flexible model (4) which did not incorporate the population hazard *λ_P_*, in order to obtain an estimate of the overall recurrence-free mortality hazard. As shown in Figure 2, the overall mortality hazard never reached zero; this led to the conclusion of a persistent risk of mortality. With our new flexible model (4), we observed that curative surgery generates an excess death due to post-surgical complication only during a few months after surgery.

Then, in the third step, the tests of common covariate effects on the events "local recurrence" and "distant metastasis" were not significant (p = 0.58, p = 0.94, and p = 0.07 for sex, age, and stage, respectively). The estimated hazard ratios are shown in Table 1. The effect of sex was statistically significant on recurrence events (either local or distant) and protective for women but was not significant on the excess death event. The effect of age at diagnosis on recurrence events was not significant but it was significant on excess-death event, old patients having poorer prognoses than young ones. Cancer stage had the same strong significant effect on local and distant recurrence events. The effect of cancer stage on the excess-death event was also statistically significant.

**Table 1:** Results of the analysis of 936 French colon cancer patients: Adjusted Hazard Ratios for covariates sex, age and stage of cancer at diagnosis associated with the events local recurrence, distant metastasis and excess death, with the 95% confidence interval.

Type of event and Covariate	Adjusted Hazard Ratio with 95% confidence interval	p-value for a 2-tailed Wald test
Local recurrence		
Man	1	
Woman^#^	0.71 [ 0.53; 0.94 ]	0.02
Age^#^	1.00 [ 0.99; 1.01 ]	0.55
Stage I	1	
Stage II^#^	3.50 [ 2.11; 5.80 ]	< 0.01
Stage III^#^	7.36 [ 4.50; 12.05 ]	< 0.01
Distant metastasis		
Man	1	
Woman^#^	0.71 [ 0.53; 0.94 ]	0.02
Age^#^	1.00 [ 0.99; 1.01 ]	0.55
Stage I	1	
Stage II^#^	3.50 [ 2.11; 5.80 ]	< 0.01
Stage III^#^	7.36 [ 4.50; 12.05 ]	< 0.01
Excess death		
Man	1	
Woman	0.77 [ 0.42; 1.42 ]	0.74
Age	1.12 [ 1.09; 1.16 ]	< 0.01
Stage I	1	
Stage II	1.48 [ 0.73; 2.98 ]	0.05
Stage III	2.76 [ 1.39; 5.48 ]	0.02

^# ^The same effect was estimated on local recurrence event and distant metastasis event.

## Discussion

To our knowledge, model (3) and its flexible refinement model (4) proposed here are the first to consider competing risks within the framework of the excess hazard regression model. These new models make it possible to estimate (i) the hazard function for each type of pre-specified event, including the recurrence-free excess death hazard function, (ii) changes over time in their ratio, and (iii) the effect of covariates on the hazard of each event, including the excess death event. Furthermore, the joint estimation of all parameters allows comparisons between covariate effects associated with different types of events in a single analysis. Analysis of the population-based dataset on French colon cancer patients using model (4) underlines the importance to model in a flexible way the ratio of the baseline hazards of the events and permits new insights into the benefit of surgery.

A joint modelling of the hazards allows fitting models with common parameters; this results in more parsimonious models and more efficient parameter estimators [4,18]. This may be interesting when studying related events (such as local recurrence and distant metastasis in cancer patients) to allow some covariates to have the same effect. The simultaneous estimation on duplicated data facilitates direct comparisons between event-specific baseline hazards and also between covariate effects, using standard statistical tests. Tai et al. compared the joint modelling of event-specific hazards to other non-parametric approaches commonly used in competing risk situations [27]. Their comparisons were based on graphical representations of the cumulative incidence function (i.e., the probability for a specific event occurring before a given time *t*) and they have shown that the estimates of the cumulative incidence functions obtained with the joint modelling of hazards were very close to the non-parametric estimates [27].

In our new model (3), we assumed a common pattern for all event-specific hazards, which may be dubious in most cases. Whenever the assumption of a common pattern does not hold, a simple approach could be to analyse the competing risk data stratified on the type of event. While this approach assigns different baseline hazard functions for each type of event, it does not allow comparisons between all types of events in a single analysis. In our new flexible model (4), the introduction of a time-dependent log HRs *b_k_*(*t*) between event-specific hazards offers the advantage of relaxing the assumption of a common pattern and of estimating jointly the baseline hazards relative to all types of events. As shown in our colon cancer application, this helps understanding the natural history of the disease through the way the event-specific baseline hazard changes over time, including the recurrence-free excess death hazard. Moreover, a parametric framework allows using classical and well-known inference tools: for example, comparing the model with a constant *b_k _*against the model with *b_k_*(*t*) using the LR test with the appropriate df allows to test the assumption of common pattern for baseline hazard functions.

The present work focuses on modelling the event-specific hazard which is not directly interpretable as the marginal hazard function. Indeed, interpreting the event-specific hazard as the marginal function would be equivalent to assuming independence between competing events, whereas this assumption cannot be met [4,10,18,32]. Thus, the HR for the effect of a covariate, estimated using either the Lunn-McNeil model or the above-shown models (3) and (4), is interpretable as an event-specific HR; however, caution must be exercised when interpreting it in terms of marginal probability. Under the assumption of independence between different types of events, the marginal probability describes the time to event distribution in a hypothetical situation where no competing events are assumed to occur [4,10,33]. An alternative approach for studying competing risks is to model the subdistribution hazard function [14], which permits estimating covariate effects that are directly interpretable in terms of marginal probabilities.

Regression splines have been widely used in classical survival studies [25,34-36] and in excess hazard models [21,23]. The main advantage of regression splines is the possibility to model several kinds of patterns while being linear in the parameters. Besides, cubic regression splines offer a good compromise between flexibility and smoothness [34]. However, the location of the knots needs a careful consideration. Knots may be fixed 'a priori' whenever the shape of the hazard function is available. When nothing is known about the hazard function, the knots may be specified using data-dependent criteria, according to the empirical distribution of the observed times to events. In our simulations and application, the knots for cubic regression splines were chosen 'a priori'. In colon cancer, a high excess death is often observed during the first year [21,37]. Cubic regression splines may also be used to model and to test non-linear effects of covariates on event-specific hazard functions in a simple way: since any model with a linear effect of a given covariate is nested within models with a non-linear effect of the same covariate modelled using regression splines, a simple LR test may be used. Furthermore, when the PH assumption does not hold for some covariates, the time-dependent effects of these covariates may be introduced in the flexible model (4) in the same manner as time-dependent HRs between event-specific hazard functions. Therefore, model (4) may be extended to estimate adjusted effects of HRs between event-specific hazard functions and adjusted effects of covariates assuming time-dependent effects as well as constant effects.

The data on colon cancer patients showed an important excess death that occurred just after surgery but decreased thereafter to become null a few months later. We have shown that if the expected mortality hazard is not taken into account, the overall mortality hazard will be more important and never reach zero. Local recurrence and distant metastasis hazards reached peaks nearly one year after diagnosis and then decreased slowly, confirming the importance of keeping patients under close medical supervision [5,38,39]. The effect of sex was identical on the three events, men having poorer prognoses than women and this effect was advantageously modelled using our model with a (single) common parameter rather than three.

In this work, we limited the analysis to the first occurring event but other recurrences, as well as death after a recurrence, may be observed. An interesting future work, based on the idea of our new model, would be to study all times to events as multivariate failure-time data, including an unmeasured "frailty" term to take into account the correlation between times to events, such as that between the time to distant metastasis and the time to excess death [40,41].

## Conclusions

The new models proposed in this paper allow considering competing risks within the framework of excess hazard regression model. They make it possible to estimate in a flexible way the hazard function for each type of pre-specified events, including the recurrence-free excess death hazard function. A joint estimation of all parameters allows direct comparison between covariate effects and may provide more parsimonious models and more efficient parameter estimators.

## List of abbreviations

PH: proportional hazard; LR: Likelihood Ratio; HR: Hazard Ratio; AIC: Akaike Information Criterion; df: degree of freedom;

## Competing interests

The authors declare that they have no competing interests.

## Authors' contributions

AB and RG conceived the study, performed the statistical analysis and drafted the manuscript. LR, GL and VJ conceived the study and helped drafting the manuscript. All authors read and approved the final manuscript.

## Pre-publication history

The pre-publication history for this paper can be accessed here:

http://www.biomedcentral.com/1471-2288/11/78/prepub

## Supplementary Material

Additional file 1 **Data generation and design of simulation studies**. Detailed presentation of the way data are generated and simulation are carried out (see also references [42-45]) with numerical and graphical results on the performance of the estimators. Click here for file
