# Supplementary material for: Competing risk models to estimate the excess mortality and the first recurrent-event hazards
Source: BMC Med Res Methodol. 2011 May 25;11:78. doi: 10.1186/1471-2288-11-78 (PMC3123657; doi:10.1186/1471-2288-11-78)
Supplement: Additional file 1 — Data generation and design of simulation studies. Detailed presentation of the way data are generated and simulation are carried out (see also references [42-45]) with numerical and graphical results on the performance of the estimators. [file 1471-2288-11-78-S1.PDF]

# Additional file 1

## Title: Data generation and design of simulation studies

## Description: Detailed presentation of the way data are generated and simulation are carried out (see also references [42-45]) with numerical and graphical results on the performance of the estimators

Simulation studies were conducted to assess the performance of the estimators obtained from the flexible model (4) in the case of 3 competing events of whom death. The times to events $T_1$, $T_2$ and $T_3$, were supposed to depend on three independent prognostic factors: sex, age at diagnosis (as continuous covariate), and X, a binary covariate of interest. Sex was generated with P(male) = P(female) = 0.5. The age at diagnosis was generated so as to represent approximately the empirical distribution of colon cancer in French registries [42]: 25% of patients aged 30-64 years, 35% aged 65-74 years, and 40% aged 75 years and more. Covariate X was generated from a binary distribution with P(X=0) = P(X=1) = 0.5.

In agreement with Le Teuff [42], we considered that the time to death (event $T_3$) is the minimum of two distinct times to death: one due to “excess death”, $T_{3+}$, and another due to “population hazard”, $T_{3P}$ (Thus, $T_3$=min($T_{3+}$,$T_{3P}$)). The times to events $T_1$, $T_2$, and $T_{3+}$ were generated from three independent generalized Weibull distributions [43,44] using the inverse transform method [45] with distinct parameters for each event. Moreover, for each subject, $T_{3P}$ was calculated using the French national vital statistics published by the Institut National de Statistique et des Études Économiques (INSEE) based on sex and age. In all simulations, the covariate effects on each event-specific hazard function were assumed proportional. The effects of the covariates on the times to events $T_1$, $T_2$, and $T_{3+}$ were respectively equal to ln(0.8), ln(1.2), and ln(1.2) for sex; ln(1.03), ln(1.03), and ln(1.05) for age; and ln(2), ln(3), and ln(1) for X.

A censoring time $C$ resulting from a random drop-out mechanism was generated from a uniform distribution U[0, $a$], where $a$ was selected so as to obtain approximately a pre-specified drop-out rate. In addition, administrative censoring was introduced at $C_A$= 7 years, time at which all subjects still at risk were censored. Finally, the observed time $T$ was determined as the minimum of $(T_1, T_2, T_3, C, C_A)$ and the data were constructed so as to indicate if the subject was censored at $T$ or not and, in the latter case, to account for the type of event that occurred at $T$.

In order to evaluate the method under different censoring patterns and different sample sizes, we considered three rates of drop-out censoring (0%, 15%, and 30%) and two different sample sizes (a moderate sample size of 400 patients and a more important one of 1000 patients), defining thus six different scenarios. In each scenario, we generated 1000 independent random samples and all those samples were independently analyzed using the new model (4).

The statistical criteria used to evaluate the performance of the regression coefficient estimators were: (i) the relative bias (RB); i.e., the difference between the empirical mean of the 1000 parameter estimates and the true parameter $\beta$ . divided by $\beta$; (ii) the empirical coverage rate (ECR); i.e., the proportion of samples in which the 95% confidence interval includes $\beta$; (iii) the empirical standard deviations (SD) of the parameter estimates; and (iv) the mean of the 1000 estimated standard errors (SEM) of the parameter estimates. Moreover, the mean of the estimates of the baseline hazard functions were presented graphically with the 95% empirical variation at 1, 3, and 5 years.

Table S1. Results of a simulation study obtained with model (4) based on 1000 samples, with patient number N equal to 400 or to 1000 and different drop-out censoring levels (0%, 15%, and 30 %)

| | Covariates | 0% of drop-out censoring level | | | 15% of drop-out censoring level | | | 30% of drop-out censoring level | | |
|---|---|---|---|---|---|---|---|---|---|---|
| | (True HR) | RB | ECR | SD (SEM) | RB | ECR | SD (SEM) | RB | ECR | SD (SEM) |
| N=400 | | | | | | | | | | |
| Type 1 event | | | | | | | | | | |
| | Sex ( 0.8 ) | -0.001 | 94.6 | 0.225 (0.226) | 0.001 | 93.6 | 0.261 (0.250) | 0.035 | 94.4 | 0.295 (0.281) |
| | Age ( 1.03 ) | 0.006 | 95.0 | 0.0075 (0.0075) | 0.014 | 94.7 | 0.0084 (0.0083) | 0.013 | 94.1 | 0.0096 (0.0093) |
| | X ( 2 ) | 0.032 | 95.0 | 0.229 (0.230) | 0.029 | 94.2 | 0.262 (0.255) | 0.036 | 94.9 | 0.293 (0.288) |
| Type 2 event | | | | | | | | | | |
| | Sex ( 1.2 ) | 0.004 | 94.3 | 0.243 (0.237) | 0.044 | 94.3 | 0.270 (0.266) | 0.025 | 94.6 | 0.313 (0.307) |
| | Age ( 1.03 ) | 0.014 | 95.7 | 0.0079 (0.0080) | 0.016 | 93.5 | 0.0092 (0.0089) | 0.035 | 94.1 | 0.0106 (0.0102) |
| | X ( 3 ) | 0.019 | 94.4 | 0.259 (0.254) | 0.04 | 92.9 | 0.308 (0.287) | 0.05 | 93.9 | 0.353 (0.334) |
| Type 3 event | | | | | | | | | | |
| | Sex ( 1.2 ) | -0.039 | 93.4 | 0.154 (0.145) | -0.008 | 93.9 | 0.166 (0.155) | 0.017 | 93.5 | 0.177 (0.167) |
| | Age ( 1.05 ) | 0.033 | 91.8 | 0.0059 (0.0054) | 0.04 | 92.8 | 0.0062 (0.0058) | 0.039 | 92.8 | 0.0066 (0.0062) |
| | X ( 1 )[a] | - | 92.5 | 0.156 (0.148) | - | 94.7 | 0.159 (0.157) | - | 95.5 | 0.173 (0.168) |
| N=1,000 | | | | | | | | | | |
| Type 1 event | | | | | | | | | | |
| | Sex ( 0.8 ) | 0.018 | 96.3 | 0.138 (0.141) | -0.017 | 94.9 | 0.158 (0.155) | -0.022 | 94.5 | 0.176 (0.174) |
| | Age ( 1.03 ) | -0.001 | 96.7 | 0.0045 (0.0047) | 0.014 | 95.4 | 0.0052 (0.0052) | 0.014 | 94.5 | 0.0058 (0.0058) |
| | X ( 2 ) | 0.001 | 95.3 | 0.141 (0.143) | 0.017 | 95.3 | 0.160 (0.159) | 0.021 | 94.4 | 0.182 (0.178) |
| Type 2 event | | | | | | | | | | |
| | Sex ( 1.2 ) | 0.021 | 93.8 | 0.152 (0.148) | -0.001 | 94.8 | 0.164 (0.165) | 0.007 | 94.3 | 0.194 (0.189) |
| | Age ( 1.03 ) | 0.008 | 94.3 | 0.0052 (0.0050) | 0.007 | 94.7 | 0.0057 (0.0056) | 0.010 | 94.6 | 0.0063 (0.0064) |
| | X ( 3 ) | 0.009 | 95.1 | 0.160 (0.158) | 0.004 | 94.9 | 0.184 (0.177) | 0.005 | 94.7 | 0.211 (0.205) |
| Type 3 event | | | | | | | | | | |
| | Sex ( 1.2 ) | -0.037 | 94.4 | 0.095 (0.092) | -0.043 | 94.4 | 0.101 (0.098) | -0.047 | 95.4 | 0.107 (0.105) |
| | Age ( 1.05 ) | 0.024 | 92.0 | 0.0036 (0.0034) | 0.022 | 94.3 | 0.0036 (0.0036) | 0.022 | 94.0 | 0.0039 (0.0039) |
| | X ( 1 )[a] | - | 92.6 | 0.101 (0.093) | - | 93.7 | 0.105 (0.099) | - | 94.5 | 0.110 (0.106) |

Abbreviations: HR: hazard ratio; RB: relative bias; ECR: empirical coverage rate; SD: empirical standard deviation; SEM: standard errors mean of the 1,000 estimated parameters.

[a] The true HR of covariate X on event of type 3 was equal to 1, so RB was not defined but replaced by "-".

**Simulation results**

As shown in Table S1 (columns 3, 6, and 9), the RBs were close to zero whatever the sample size and the drop-out censoring level (range: -0.047 to 0.05). As expected, the RB increased with the drop-out censoring level for most of the parameter estimates and the impact of the drop-out censoring level was more important when N=400. Whatever the sample size and the drop-out censoring level, the ECRs were close to the nominal level of 95% (Table S1, columns 4, 7, 10), even if the ECR was slightly smaller than 95% for the parameter estimates on the hazard function of event 3. In this situation, the SEMs were under-estimated compared to the SDs (Table S1, columns 5, 8 and 11: the SEMs for covariates sex, age, and X were respectively 0.145, 0.0054, and 0.148 while the SDs were respectively 0.154, 0.0059, and 0.156). Interestingly, the ECR relative to the effect of covariate X (which was not a contributor to the expected mortality) on the hazard function of event 3 increased as the drop-out censoring level increased. Generally, the SEMs of the parameters were close to the empirical SDs.

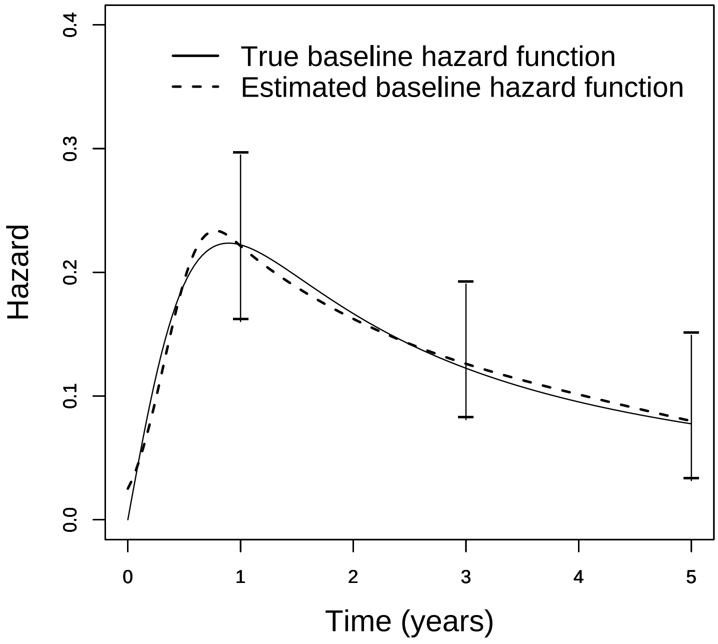


**Figure S1**

The graphed results show the accuracy of the estimates of the time-dependent functions. The mean of the estimates of the baseline hazard function of event 1, $\lambda_1(t)$, was close to the true baseline hazard function (Figure S1). The means of the estimates of the baseline hazard functions of event 2, $\lambda_2(t)$, (Figure S2a) and of event 3, $\lambda_3(t)$, (Figure S2c) were similar to their true baseline hazard functions. This similarity was also the case for the time-dependent HR relative to event 2, $\exp(b_2(t))$, (Figure S2b) and event 3 (excess death), $\exp(b_3(t))$ (Figure S2d). The empirical variations, obtained with percentiles 2.5 and 97.5 of the empirical distribution of the baseline hazard function or of the time-dependent HR, were also displayed at 1, 3, and 5 years (Figures S1 and S2). We observed that, whatever the event considered for analysis, the 95% empirical variations of the time-dependent HR were more important than the empirical variations of the baseline hazard function. This is due to the fact that the ratio of two hazard functions reflects the empirical variations of each hazard function.

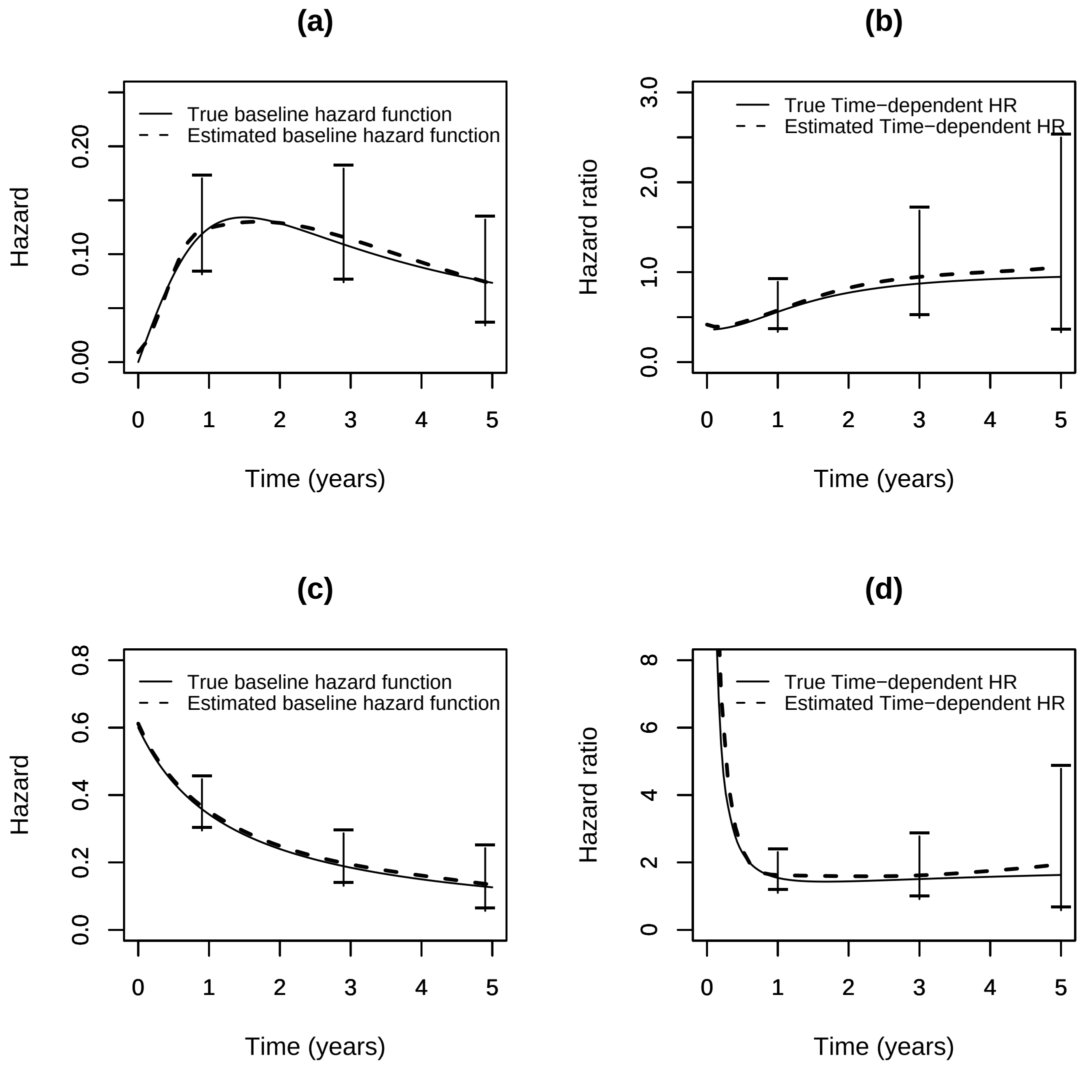

(a)
(b)
(c)
(d)
True baseline hazard function
Estimated baseline hazard function
True Time−dependent HR
Estimated Time−dependent HR
Hazard
Hazard ratio
Time (years)
0.00
0.10
0.20
0.0
1.0
2.0
3.0
0.2
0.4
0.6
0.8
0
2
4
6
8
1
3
5


**Figure S2**

# Legends to the figures

**<u>Figure S1.</u> True baseline hazard function (solid line) versus the mean of 1000 estimates of the baseline hazard function (dashed line) for event of type 1, $\lambda_1(t)$, with the 95% empirical variations at 1, 3, and 5 year. Situation with sample size = 1000 and 15% drop-out censoring level.**

**<u>Figure S2.</u> Representation of the true baseline hazard function (solid lines) versus the mean of 1000 estimates of the baseline hazard function (dashed lines) (a) for event of type 2, $\lambda_2(t)$, and (c) for event of type 3, $\lambda_3(t)$, and the corresponding true time-dependent hazard ratio versus the mean of 1000 estimates of the time-dependent hazard ratio (b) for event of type 2, $\exp(b_2(t))$, and (d) for event of type 3, $\exp(b_3(t))$. Vertical bars represent the 95% empirical variations at 1, 3 and 5 years. Situation with sample size = 1000 and 15% drop-out censoring level.**
